# Amygdala atrophies in specific subnuclei in preclinical Alzheimer's disease

**DOI:** 10.1002/alz.14235

**Published:** 2024-09-10

**Authors:** Yasmine Salman, Thomas Gérard, Lara Huyghe, Lise Colmant, Lisa Quenon, Vincent Malotaux, Adrian Ivanoiu, Renaud Lhommel, Laurence Dricot, Bernard J. Hanseeuw, for the Alzheimer's Disease Neuroimaging Initiative

**Affiliations:** ^1^ Louvain Aging Brain Lab Institute of Neuroscience UCLouvain Brussels Belgium; ^2^ Nuclear Medicine Department Saint‐Luc University Hospital Brussels Belgium; ^3^ Neurology Department Saint‐Luc University Hospital Brussels Belgium; ^4^ Department of Psychiatry Massachusetts General Hospital Harvard Medical School Boston Massachusetts USA; ^5^ WELBIO Department WEL Research Institute Wavre Belgium; ^6^ Department of Radiology Massachusetts General Hospital Harvard Medical School Boston Massachusetts USA

**Keywords:** Alzheimer's disease, amygdala subnuclei, AV1451, biomarker, florquinitau, flortaucipir, MK6240, preclinical AD, structural magnetic resonance imaging, tauopathy, tau‐positron emission tomography

## Abstract

**INTRODUCTION:**

Magnetic resonance imaging (MRI) segmentation algorithms make it possible to study detailed medial temporal lobe (MTL) substructures as hippocampal subfields and amygdala subnuclei, offering opportunities to develop biomarkers for preclinical Alzheimer's disease (AD).

**METHODS:**

We identified the MTL substructures significantly associated with tau‐positron emission tomography (PET) signal in 581 non‐demented individuals from the Alzheimer's Disease Neuroimaging Initiative (ADNI‐3). We confirmed our results in our UCLouvain cohort including 110 non‐demented individuals by comparing volumes between individuals with different visual Braak's stages and clinical diagnosis.

**RESULTS:**

Four amygdala subnuclei (cortical, central, medial, and accessory basal) were associated with tau in amyloid beta‐positive (Aβ+) clinically normal (CN) individuals, while the global amygdala and hippocampal volumes were not. Using UCLouvain data, we observed that both Braak I‐II and Aβ+ CN individuals had smaller volumes in these subnuclei, while no significant difference was observed in the global structure volumes or other subfields.

**CONCLUSION:**

Measuring specific amygdala subnuclei, early atrophy may serve as a marker of temporal tauopathy in preclinical AD, identifying individuals at risk of progression.

**Highlights:**

- Amygdala atrophy is not homogeneous in preclinical Alzheimer's disease (AD).
- Tau pathology is associated with atrophy of specific amygdala subnuclei, specifically, the central, medial, cortical, and accessory basal subnuclei.
- Hippocampal and amygdala volume is not associated with tau in preclinical AD.
- Hippocampus and CA1‐3 volume is reduced in preclinical AD, regardless of tau.

## 1 BACKGROUND

Alzheimer's disease (AD) is a progressive neurodegenerative disorder characterized by the abnormal aggregation of amyloid beta (Aβ) and hyperphosphorylated tau protein, resulting in neurodegeneration, progressive cognitive decline, and, ultimately, dementia.^1^, ^2^ Aβ and tau pathologies are detectable in clinically normal (CN) individuals, indicating a preclinical AD stage.^3^, ^4^ Based on autopsy^5^, ^6^ and imaging^7^ studies, the prevailing research hypothesis posits that Aβ precedes and exacerbates tau pathology,^8^ which in turn leads to neurodegeneration in specific brain regions and corresponding cognitive deficits.

Detecting the topography of protein aggregates requires positron emission tomography (PET), a technique sparsely available outside of academic centers. Magnetic resonance imaging (MRI) is currently cheaper, more available, and less invasive than tau‐PET.^9^ While MRI can highlight brain atrophy in AD, global brain atrophy is not specific to AD pathology. Studying the association between early tau deposition and atrophy in specific brain areas is therefore of particular interest to identify brain regions whose atrophy could be specific to tau pathology related to AD and ultimately detect individuals who will develop AD symptoms in the future.^10^

Tau pathology initially manifests in the medial temporal lobe (MTL), first in the transentorhinal cortex (Braak stage I), then in the hippocampus and the amygdala (Braak stage II),^11^, ^12^ leading to neuronal loss. However, MTL atrophy is not only observed in AD,^13^, ^14^ as evidenced by the recent description in limbic‐predominant age‐related TAR DNA‐binding protein 43 (TDP‐43) encephalopathy (LATE).^15^, ^16^ Autopsy studies have demonstrated that MTL substructures, that is, hippocampal subfields^17^, ^18^ and amygdala subnuclei,^19^ have variable degrees of tau burden.^11^, ^12^ So far, though, the association between in vivo tau pathology and substructures of the MTL, specifically amygdala nuclei, has not been studied.

Hippocampal subfield atrophy have been measurable using MRI automated segmentation methods since the 2010s.^20^, ^21^, ^22^ Atrophy of subiculum and cornu Ammonis (CA) 2‐3 was initially observed in mildly cognitively impaired individuals using FreeSurfer version 5.^23^ Subsequent work identified presubiculum atrophy as most associated with cognitive decline in preclinical AD.^24^ The exact subfields most associated with tau pathology, as measured using PET, are still to be determined. Until recently, segmenting amygdala subnuclei was not feasible using automated MRI pipelines. The literature on AD neuroimaging research is therefore very sparse, although a few studies have investigated amygdala subnuclei atrophy based on clinical diagnosis^25^, ^26^ but without investigating the association between volume and tau pathology. Yet data indicate the amygdala as the structure displaying the highest density of tau tangles in preclinical AD.^27^, ^28^

We aimed to leverage the release of FreeSurfer version 7.2, which allows for segmenting both hippocampal subfields^29^ and amygdala subnuclei,^30^ to highlight the MTL substructures displaying significant associations between tau‐PET signal and brain volumetry in preclinical AD. Subsequently, we evaluated whether the volume of the identified structures was reduced in an independent cohort of preclinical AD and further reduced in prodromal AD.

## 2 METHODS

### 2.1 Participants

#### 2.1.1 Alzheimer's disease neuroimaging initiative

Part of the data used in the preparation of this article was obtained from the Alzheimer's Disease Neuroimaging Initiative (ADNI) database (adni.loni.usc.edu). The ADNI was launched in 2003 as a public–private partnership, led by Principal Investigator Michael W. Weiner, MD. The primary goal of ADNI has been to test whether serial MRI, PET, other biological markers, and clinical and neuropsychological assessment can be combined to measure the progression of mild cognitive impairment (MCI) and early AD. For up‐to‐date information, see www.adni-info.org.

Non‐demented participants in the ADNI‐3 cohort with available T1‐weighted MRI, [^18^F]Florbetapir Aβ‐PET, and [^18^F]AV1451 tau‐PET data done within the same year were included in this study. ADNI participants were classified as CN if they had a Mini‐Mental State Examination (MMSE) score of ≥24, a Clinical Dementia Rating (CDR) score of 0, and a normal memory function documented by scoring above education‐adjusted cutoffs on the Logical Memory II subscale (Delayed Paragraph Recall, Paragraph A only) from the Wechsler Memory Scale–Revised (≥9 for 16 or more years of education; ≥5 for 8 to 15 years of education; ≥3 for 0 to 7 years of education). They were classified as having MCI if they had a MMSE score of ≥24, a CDR score of 0.5, and an abnormal memory function documented by scoring below education‐adjusted cutoffs on the Logical Memory II from the Wechsler Memory Scale–Revised (<11 for 16 or more years of education; ≤9 for 8 to 15 years of education; ≤6 for 0 to 7 years of education). Participants presetting any psychiatric or neurological condition (except suspected AD in case of MCI) or any significant systemic illness were excluded from the ADNI‐3 study.

A summary standardized uptake value ratio (SUVR) from [^18^F]Florbetapir PET (publicly available in processed data on the ADNI website) determined each participant's Aβ status using a threshold of SUVR ≥ 1.11,^31^ corresponding approximately to a Centiloid threshold of >20. The Centiloid Aβ‐PET scale is anchored at 0 and 100 Centiloids, with a 0 Centiloid score reflecting a definitively Aβ‐negative brain (originally calculated as the average value of a group of healthy subjects below the age of 45) and 100 Centiloids reflecting the average signal observed in patients with typical mild or moderate AD dementia.^32^ This harmonized method (originally anchored to [11C] PiB but now applicable to [18F] tracers after calibration) has great potential to produce cohesive and comparable results from disparate clinics and cohorts across the world as it makes it possible to transform resulting PET signal measures obtained with different radiotracers into a common unit.^33^ In total, 581 participants were selected and categorized into Aβ‐negative (Aβ−) CN individuals (*n* = 305), Aβ‐positive (Aβ+) CN (*n* = 144), and Aβ+ MCI (*n* = 132). Aβ− MCI (*n* = 141) were not included. Data download was conducted on September 1, 2023. Data collection was conducted between January 2017 and January 2023.

#### 2.1.2 UCLouvain

Non‐demented older adults were enrolled in a study based at UCLouvain, Brussels, Belgium. Patients were recruited through the memory clinic of the hospital (Cliniques Universitaires Saint‐Luc) while volunteers were recruited from other studies though mailbox announcements in the hospital's neighborhood. Volunteers were selected from this pool, enriching the apolipoprotein E (*APOE*) ε4 carriers to match the observed frequency of *APOE* carriage in patients. Exclusion criteria for both patients and volunteers included focal brain lesions, epileptic seizures, major depression or psychiatric conditions, and alcohol or drug abuse. Informed consent was obtained from all individual participants in accordance with the principles of the Declaration of Helsinki. Ethical approval for the study was granted by the Ethics Committee of UCLouvain (Date: May 13, 2019; Eudra‐CT number: 2018‐0034/73‐94).

**RESEARCH IN CONTEXT**

1. **Systematic review**: The amygdala and hippocampus are early affected by tau pathology in Alzheimer's disease (AD). Few studies have investigated the atrophy of specific amygdala subnuclei and hippocampal subfields, and none have investigated the relation to tau pathology in preclinical AD. We reviewed the existing literature on PubMed Central for imaging studies of amygdala subnuclei or hippocampal subfields.
2. **Interpretation**: We demonstrated that only the central, cortical, medial, and basal accessory nuclei of the amygdala are associated with tauopathy in preclinical AD and atrophic from Braak stage I‐II, suggesting that they could serve as early MRI markers.
3. **Future directions**: Further studies of amygdala subnuclei atrophy using higher resolution and studies including biomarkers of other proteins associated with amygdala or hippocampal atrophy, such as TDP‐43, are essential to better understand the differential involvement of medial temporal substructures in preclinical AD.

Participants were categorized using their clinical and Aβ status (bioclinical status), as in the ADNI cohort.^34^, ^35^, ^36^, ^37^ Each participant underwent a neuropsychological assessment evaluating four cognitive domains: memory (Free and Cued Selective Reminding Test, French version), language (Lexis Naming Test, the Category Fluency Test for animals, and the Letter Fluency Test for the letter “P”), executive functions (Trail Making Test, Luria's Graphic Sequences [adaptations in French]), and the visuospatial functions (Clock Drawing Test and the Praxis part of the Consortium to Establish a Registry for Alzheimer's Disease [CERAD] battery). Each domain was assessed based on three measures that were used to compute a *z*‐score (for further details about cognitive testing, see Ivanoiu et al. 2015).^34^ A cognitive domain was considered impaired if the corresponding *z*‐score fell below −1.5 SD compared to the mean performance of an independent sample (composed of 32 CN individuals who remained cognitively stable over an 8‐year period). Participants were classified as having MCI based on a MMSE score ≥24/30 and at least one impaired cognitive domain, or being CN when the MMSE ≥24/30 and performance on all four domains was above −1.5 SD. Aβ status was determined either by lumbar puncture or Aβ‐PET using either [^18^F]Flutemetamol (Vizamyl GE Healthcare) or [^11^C]PiB. In cerebrospinal fluid (CSF), amyloid‐β42 (Aβ42) and phosphorylated tau at threonin^181^ were measured using Lumipulse automated assays. Aβ‐PET quantification method followed the Centiloid Aβ‐PET scale methods as already described in previous work.^35^ It uses the same reference region (whole cerebellum) and data processing as the Centiloid method used for ADNI data. Aβ status was deemed positive if at least one of these three conditions was met: Centiloid > 20; CSF Aβ42 < 437pg/mL; or CSF Aβ42 < 650pg/mL and CSF P‐tau > 61pg/mL.^38^ Twelve participants had both lumbar puncture and Aβ‐PET. When results were discordant (*n* = 2), we defined Aβ status using the PET result.

In total, 110 participants were included and categorized into bioclinical classification based on Aβ status and neuropsychological evaluation and including Aβ− CN individuals (*n* = 44), Aβ+ CN (*n* = 18), and Aβ+ MCI (*n* = 48).

### 2.2 Imaging acquisition

#### 2.2.1 ADNI

##### MRI

Three‐dimensional (3D) T1‐weighted MRI scans in ADNI were acquired using imaging protocols detailed in prior publications.^39^, ^40^

##### [^18^F]Florbetapir Amyloid PET

Aβ‐PET imaging consisted of a continuous 20‐min brain scan (four frames lasting 5 min) administered 50 min after an injection of 10mCi of [^18^F]Florbetapir. Centiloid data from Aβ‐PET were downloaded from the ADNI data archive. [Correction added on October 5, 2024, after first online publication: Heading has been modified from ‘[^18^F]AV‐1451 Tau‐PET’ to ‘[^18^F]Florbetapir Amyloid PET’.]

##### [^18^F]AV‐1451 Tau‐PET

Tau‐PET imaging involved a continuous 30‐min brain scan (six frames of 5‐min duration) administered 75 min after injecting 10mCi of [^18^F]Flortaucipir (AV1451). Postprocessed PET images were generated by averaging co‐registered individual frames, reoriented in a standardized image space to align the anterior–posterior axis with the AC–PC line. Subsequently, scanner‐specific filtering was applied to generate an image with a uniform isotropic resolution of 8 mm full width at half maximum. Further details on the preprocessing method used can be found at http://adni.loni.usc.edu/methods/pet‐analysis‐method/pet‐analysis/.

#### 2.2.2 UCLouvain

##### MRI

3D T1‐weighted MRI sequences were obtained using a 3 Tesla MRI (GE Signa Premier 3T, GE Healthcare, Chicago IL) equipped with a 48‐channel phased‐array head coil. Two distinct acquisition processes were employed, with acquisition no. 1 used for the first 33 subjects who had a 3DT1 MRI acquired in a European study that was not extended throughout the present study.

Acquisition no. 1: 196 slices were acquired using the following parameters: repetition time (TR) = 7.2 ms, echo time (TE) = 2.9 ms, flip angle (FA) = 11°, slice thickness 1.2 mm; field of view (FOV) 270 × 270 mm^2^, acquisition matrix 256 × 256, acquired voxel size = 1.05 × 1.05 × 1.2 mm^3^.

Acquisition no. 2: 156 slices were acquired using the following parameters: TR = 2188 ms; TE = 2.9 ms; inversion time = 900 ms (MPRAGE), F = 8°; slice thickness = 1 mm; FOV = 256 × 256 mm^2^, acquisition matrix = 256 × 256; acquired voxel size = 1 × 1 × 1 mm^3^. [Correction added on October 5, 2024, after first online publication: In the preceding sentence, ‘TE = 2.9 m’ has been modified to “TE = 2.9 ms”.]

When available (*n* = 100 of 110), we also used a 3D T2‐weighted MRI scan to improve the segmentation of hippocampal subfields and amygdala subnuclei. We tested whether the acquisition protocol, including the availability of a T2 sequence, impacted our results by including the acquisition protocol as a covariate in all models. Protocol acquisition did not modify any of the conclusions presented in the manuscript.

##### [^18^F]MK6240 tau‐PET

[^18^F]MK6240 (Lantheus Inc.) is an investigational drug under study as a second‐generation cerebral tau tangle imaging agent. Radiosynthesis was conducted at KULeuven and delivered to our clinic in less than an hour. Ninety minutes after intravenous administration of [^18^F]MK6240 (target activity 185 ± 5 MBq) a 30‐min dynamic list‐mode acquisition was performed on a Philips Vereos digital PET‐CT (Philips Healthcare). Images were reconstructed using the manufacturer's reconstruction algorithm, which includes attenuation, scatter, decay correction, and time‐of‐flight information. Point spread function (PSF) and 1‐mm reslicing were also computed using the manufacturer's algorithm to obtain a better resolution recovery.^37^

Two trained nuclear physicians (R.L. and T.G.) determined a visual Braak‐like stage (0 to VI) for every tau‐PET scan fused with a 3DT1 MRI for precise anatomical reference. The Braak stage assigned to a subject was the furthest Braak region where a significative signal was observed. For some intricate scans, MRI segmentation was used to assist visual reading. Based on previous work by Schöll and colleagues on Braak staging applied to brain imaging,^41^ regions were determined and grouped into categories including Braak 0 (not a single positive region, *n* = 52), Braak I‐II (positive exclusively in the MTL, *n* = 6), Braak III‐IV (positive in the temporal neocortex and/or posterior cingulate, *n* = 18), and Braak V‐VI (positive in the fronto‐parietal neocortex and/or in the occipital lobe, *n* = 34).^37^ Visual staging of tau‐PET images was not conducted in the ADNI cohort.

### 2.3 MRI analysis

All MRI scans were processed locally, with raw data from the ADNI‐3 cohort downloaded and processed in the same manner as data from the UCLouvain cohort.

**FIGURE 1:**
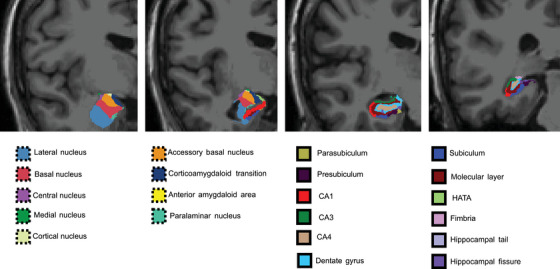
Amygdala subnuclei and hippocampus subfield segmentation. Amygdala (left, dotted) and hippocampus (right, solid) substructure segmentation from FreeSurfer version 7.2.

Subcortical segmentation and cortical parcellation of structural MRI data were performed using FreeSurfer version 7.2.^42^ First, FreeSurfer standard recon‐all steps were applied to the individual structural data of all participants. Detailed segmentation was then performed for the hippocampal subfields and the amygdala subnuclei. The hippocampal formation was divided into 12 hippocampal subfields, including parasubiculum, presubiculum, subiculum, CA1, CA3 (which includes CA2), CA4, dentate gyrus (DG), molecular layer, hippocampal amygdaloid transition area (HATA), fimbria, hippocampal tail, and hippocampal fissure.^29^ The amygdala was divided into nine subnuclei, including lateral nucleus, basal nucleus, accessory basal nucleus, medial nucleus, central nucleus, paralaminar nucleus, cortical nucleus, cortico‐amygdaloid transition, and anterior amygdala area^30^ (Figure 1).

All regions were averaged over the left and right hemispheres.

### 2.4 MRI tau‐PET coregistration

ADNI regional tau‐PET SUVR data were computed in FreeSurfer version 7 and downloaded from the ADNI data archive in the most fully postprocessed format. Up‐to‐date information about ADNI imaging protocols can be found at http://adni.loni.usc.edu/methods/pet‐analysis‐method/pet‐analysis.

Data from UCLouvain were locally processed. Tau‐PET scans were co‐registered with the corresponding T1‐weighted MRI scans using the PetSurfer pipeline, a set of tools within FreeSurfer for end‐to‐end integrated MRI‐PET analysis. In ADNI, the inferior cerebellar gray matter was used as reference region to generate Flortaucipir SUVR, whereas in UCLouvain, the entire cerebellar gray matter was used to generate MK6240 SUVR. In both cohorts, a meta‐temporal region of interest (ROI) that included the amygdala, the entorhinal, parahippocampal, fusiform, inferior temporal, and middle temporal cortices was then calculated as previously described^43^ using SUVR extracted from the Desikan‐Killiany Atlas.

### 2.5 Statistical analysis

Demographic data were compared between cohorts (ADNI vs UCLouvain) and between groups (Aβ− CN, Aβ+ CN, and Aβ+ MCI) using Mann–Whitney tests for continuous data and chi‐squared tests for dichotomous data. Given the difference in tracers used across cohorts, tau‐PET SUVR data were *z*‐scored using the mean and standard deviation of Aβ− CN before comparing cohorts.

First, an exploratory study was conducted in the Aβ+ CN group of the ADNI cohort identifying hippocampal and amygdala substructures whose volume explained at least 1% of the variance in temporal tauopathy (Spearman's *R* ^2 ^> 0.010, two‐tail *p* value ≤ 0.20). We selected a liberal threshold to only exclude substructures whose volume was not associated with early tau pathology in preclinical AD. Second, volumes of the tau‐associated substructures identified in ADNI were averaged into a substructure aggregate. Third, a partial Spearman correlation was calculated between the temporal meta‐ROI tau SUVR and the volume of global hippocampus and global amygdala. A partial Spearman correlation was also calculated between the temporal meta‐ROI tau SUVR and the substructure aggregates for each group (Aβ− CN, Aβ+ CN, Aβ+ MCI), adjusting for the global structure's volume (hippocampus or amygdala) to verify whether the association between volume and tau pathology was specific for the substructure or was explained by the global structure atrophy. We reported two‐tail *p* values. To explore the association between Aβ and volume of the MTL structures, a partial Spearman correlation was also calculated between Centiloid scale and amygdala and hippocampus substructures within the Aβ+ CN.

These results were then confirmed in an independent cohort from UCLouvain. First, partial Spearman coefficient was computed between temporal meta‐ROI tau SUVR and the tau‐associated substructures identified in ADNI adjusting for the volume of global structures. Second, we examined the volume difference in global structures, substructures, and substructure aggregate between visually determined Braak stage and the bioclinical classification using linear regressions. Post hoc contrast analyses were performed using Braak stage 0 or Aβ− CN as reference group. We used false discovery rate (FDR)‐adjusted *p* values to account for multiple comparisons.

All analyses were adjusted for age, sex, years of education, MRI protocol, and intracranial volume (ICV) and computed using R version 4.2.2 (packages ppcor and emmeans).

## 3 RESULTS

### 3.1 Characteristics of participants

The demographics of both ADNI and UCLouvain cohorts are given in Table 1. In short, Aβ+ CN and Aβ+ MCI were significantly older than Aβ− CN in both cohorts. Aβ+ CN and Aβ+ MCI had a higher proportion of *APOE* ε4 carriers in ADNI compared to Aβ− CN (not in UCLouvain given the enrichment in CN *APOE* ε4 carriers). Aβ+ MCI subjects had a significantly lower MMSE than Aβ+ CN and Aβ− CN participants. Furthermore, Aβ+ MCI had higher temporal tauopathy than Aβ+ CN, who had higher tauopathy than Aβ− CN. No difference was observed in terms of sex distribution or ICV.

**TABLE 1:** Characteristics of participants in ADNI and UCLouvain cohorts.

	ADNI			UCLouvain		
Mean ± SD	CN Aβ−	CN Aβ+	MCI Aβ+	CN Aβ−	CN Aβ+	MCI Aβ+
***N***	305	144	132	43	18	49
**Age (y)**	71.0 ± 6.9	74.5 ± 7.8 ***	74.4 ± 7.4 ***	67.8 ± 8.7 †	72.4 ± 6.4*	72.5 ± 7.0 **
**Sex (%F)**	58.7%	60.4%	47.7%	59.1%	38.9%	61.2%
**Education (y)**	16.8 ± 2.2	16.7 ± 2.3	16.1 ± 2.5*	16.9 ± 2.9†	17.2 ± 3.4	15.4 ± 3.9
***APOE* carriers**						
ε4−	64.9%	48.6%	27.3%	51.2%	50.0%	34.7%
ε4+	20.7%	40.3%	50.0%	48.8%	50.0%	65.3%
Missing	14.4%	11.1%	22.7%			
**MMSE**	29.2 ± 1.1	28.9 ± 1.3	27.3 ± 2.2 ***	28.8 ± 1.0 †	28.7 ± 1.1	26.3 ± 0.8 *** †††
**ICV (mm^3^)**	1,491,572 ± 163217	1,479,759 ± 157099	1,517,682 ± 135177	1,541,887 ± 135177†	1,503,608 ± 172117	1,468,859 ± 16144*
**Tau SUVr**	1.17 ± 0.10	1.24 ± 0.16 ***	1.48 ± 0.35 ***	0.82 ± 0.12	1.01 ± 0.26 ** †	2.22 ± 0.84 *** †††

*Note*: Demographic table summarizing general, cognitive, ICV, and tau‐PET data from ADNI and UCLouvain cohorts. Differences between the CN Aβ− group and the other groups were assessed with the Mann–Whitney test and *p* values are displayed as follows: ^*^ *p* < 0.05; ^**^ *p* < 0.01; ^***^ *p* < 0.0001. Differences between ADNI and UCLouvain subjects were assessed using the Mann–Whitney test, with *p* values displayed as follows: ^†^ *p* < 0.05; ^††^ *p *< 0.01; ^†††^ *p *< 0.0001. Given the differences in the tau‐PET tracers used, comparisons between cohorts were made using tau‐PET *z*‐scores. *Z*‐score value was based on mean and standard deviation of CN Aβ‐Tau SUVR.

Abbreviations: ADNI, Alzheimer's Disease Neuroimaging Initiative; ICV, intracranial volume; SUVR, standardized uptake value ratio.

UCLouvain Aβ+ MCI had significantly lower MMSE scores than ADNI Aβ+ MCI. Tau *z*‐scores were higher in Aβ+ CN and Aβ+ MCI from UCLouvain than the corresponding groups from ADNI, indicating that MCI had greater cognitive impairment and temporal tauopathy in our local cohort than in ADNI.

### 3.2 ADNI: Identification of MTL substructures associated with tau

**FIGURE 2:**
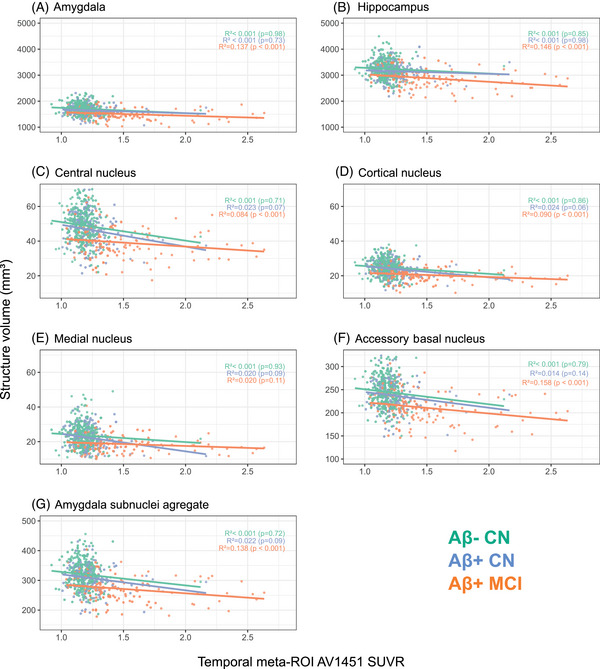
Association between volumes and temporal tau in ADNI cohort. Partial Spearman correlation analysis illustrating association between volume of medial temporal lobe structures and temporal lobe tau‐PET AV1451 SUVR in ADNI cohort. The regions considered include the amygdala, the hippocampus, and the substructures for which the *R* ^2^ was greater than 0.01 (two‐tail *p* value < 0.20) in cognitively normal Aβ+ subjects, that is, the central, cortical, medial, and accessory basal nuclei of the amygdala. An AA resulting from the combination of these tau‐associated amygdala subnuclei is also represented. Correlations are represented using the bioclinical classification. AA, amygdala aggregate; ADNI, Alzheimer's Disease Neuroimaging Initiative; SUVR, standardized uptake value ratio.

In ADNI Aβ+ CN, the global volumes of the amygdala and the hippocampus were not associated with temporal tau (amygdala: Spearman's *R* ^2^ < 0.001, two‐tail *p* value = 0.73; hippocampus: *R* ^2^ < 0.001, *p* = 0.98, adjusted for age, sex, ICV, and years of education, Figure 2, Supplementary material 1). In contrast, four MTL substructures explained at least 1% of the variance in temporal tau SUVR (two‐tail *p* < 0.20): the central (*R* ^2^ = 0.023, *p* = 0.07), cortical (*R* ^2^ = 0.024, *p* = 0.07), medial (*R* ^2^ = 0.020, *p* = 0.10), and accessory basal nuclei (*R* ^2^ = 0.014, *p* = 0.16) of the amygdala (Figure 2, Supplementary material 1). None of the hippocampal subfield volumes were significantly associated with temporal tau SUVR in ADNI Aβ+ CN.

We then grouped the identified amygdala subnuclei (central, cortical, medial, basal accessory nuclei) into an aggregate of tau‐associated amygdala substructures. This amygdala aggregate (AA) accounted for 2.2% of the variance in temporal tauopathy in the Aβ+ CN group (*p* = 0.08), a percentage that increased at 7% (*p* = 0.002) after adjusting for amygdala global volume (Figure 2, Supplementary material 1). Adding Aβ pathology in the model (as measured using the Centiloid scale) did not add explanatory power, indicating that AA atrophy was associated with tau to a greater extent than with Aβ. Such an association between temporal tauopathy and the volume of specific MTL substructures was not observed in Aβ− CN.

Unlike in CN, the volume of the global amygdala and the global hippocampus were significantly associated with temporal tauopathy in Aβ+ MCI from ADNI (amygdala: *R* ^2^ = 0.137, *p* < 0.0001; hippocampus: *R* ^2^ = 0.146, *p* < 0.0001). The AA volume was also associated with tau (*R* ^2^ = 0.138, *p* < 0.001) but did not add any additional statistical power (adjusted *R* ^2^ = 0.009, *p* = 0.28, Figure 2, Supplementary material 1). None of the hippocampal subfields or amygdala subnuclei outperformed the global hippocampal or amygdala volume to predict temporal tau in Aβ+ MCI, indicating that hippocampal and amygdala atrophy was global at the prodromal AD stage.

We repeated the analyses distinguishing right and left volume data and observed similar structures. Of note, the accessory basal, medial, and cortical nucleus correlations were driven by right‐sided atrophy, whereas the central nucleus atrophy was bilateral. We also repeated all analyses separately using the respective tau SUVR of the amygdala, entorhinal cortex, and parahippocampal cortex (using FreeSurfer ROIs) instead of the temporal meta‐ROI. We observed similar results for the four amygdala subnuclei that were systematically associated with tau burden in Aβ+ CN, justifying the use of the temporal meta‐ROI SUVR in the present study.

### 3.3 UCLouvain: Testing the association between regional tau and the volume of tau‐associated amygdala subnuclei

**FIGURE 3:**
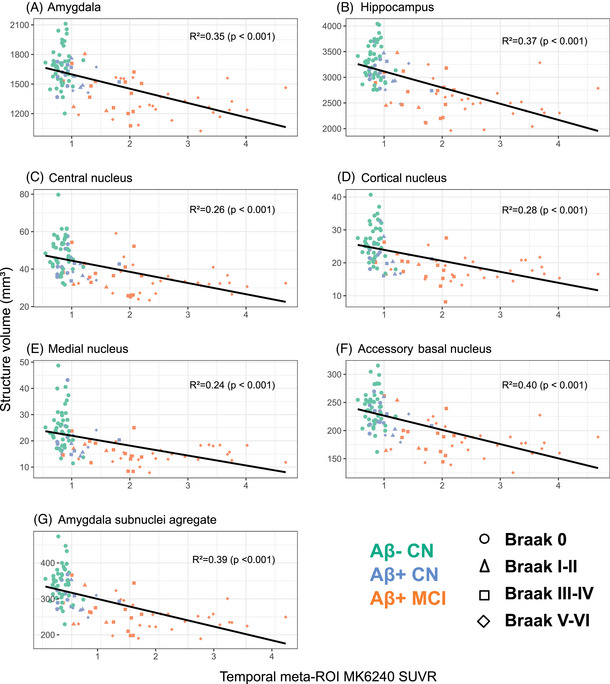
Association between volumes and temporal tau in UCLouvain cohort. Partial Spearman correlation analysis illustrating association between structure volume and temporal lobe tau‐PET MK6240 SUVR in UCLouvain cohort. Structures examined include the amygdala, hippocampus, four amygdala subnuclei (cortical, central, medial, and accessory‐basal), and the previously defined amygdala aggregate. Individual data points are discriminated by bioclinical status (color) and visual Braak stage (shape) The correlation coefficient was also adjusted for the volume of the global structure (adjusted *R* ^2^). SUVR, standardized uptake value ratio.

In UCLouvain data, we evaluated the correlation between the volume of the AA previously defined and the temporal meta‐ROI tau in Aβ+ CN (*n* = 18) as well as in all individuals (*n* = 110). In the Aβ+ CN group, the AA volume was marginally correlated with MTL tau‐PET SUVr (*R* ^2^ = 0.28, *p* = 0.090), while the global amygdala volume was not associated with MTL tauopathy (*R* = 0.06, *p* = 0.37, Supplementary material 2). In the whole cohort. Similarly, the AA volume was more strongly associated with temporal tau burden than the volume of the global amygdala (AA: *R* ^2^ = 0.39, *p* < 0.0001; amygdala: *R* ^2^ = 0.37, *p* < 0.0001, Figure 3, Supplementary material 2). After adjusting for the global amygdala volume, the volumes of AA (adjusted *R* ^2^ = 0.06, *p* < 0.0013), the cortical nucleus (adjusted *R* ^2^ = 0.03, *p* < 0.0001), medial nucleus (adjusted *R* ^2^ = 0.04, *p* = 0.05), and the accessory basal nucleus (adjusted *R* ^2^ = 0.053, *p* < 0.01) showed significant association with temporal tau.

We repeated the analyses distinguishing right and left volume data and obtained similar results. Similar results were also obtained separately using the respective SUVR of the amygdala, hippocampus, entorhinal cortex, or parahippocampal cortex instead of the SUVR of the meta‐temporal ROI.

**FIGURE 4:**
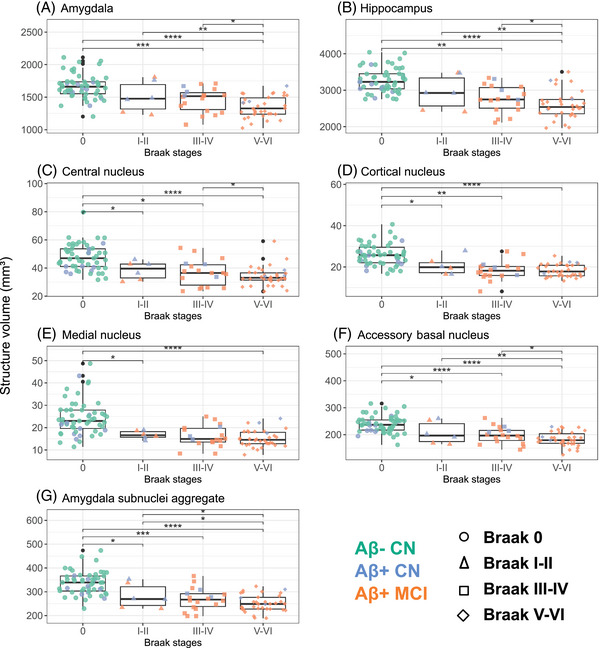
Volume difference according to visual Braak stages. Boxplots illustrate volume differences according to visual Braak stages in subjects from UCLouvain cohort. The structures examined include the amygdala, the hippocampus, four amygdala subnuclei (cortical, central, medial, and accessory‐basal nuclei), and the previously defined amygdala aggregate (AA). Individual data points are differentiated according to bioclinical status (color) and Braak visual stage (shape). Reported *p* values are *p* values adjusted for age, sex, intracranial volume, and years of education: ^*^ *p* < 0.05; ^**^ *p* < 0.01; ^***^ *p* < 0.001; ****p < 0.0001. [Correction added on October 5, 2024, after first online publication: Figure 4 has been replaced.]

As atrophy is more strongly associated with tau accumulation than with amyloid,^7^ we sought to distinguish individuals based on tau extension assessment. We used the visual Braak staging as a classification variable of our participants and investigated whether atrophy was more pronounced in the amygdala aggregate or other MTL substructures with early (Braak I‐II) or late (Braak V‐VI) tau stages. The AA, and each of its four substructures, demonstrated atrophy from Braak stage I‐II onward (AA: *d =* 1.15, *p* = 0.02; central nucleus: *d =* 0.97, *p* = 0.05; cortical nucleus: *d =* 1.09, *p* = 0.03; medial nucleus: *d =* 1.20, *p* = 0.02; accessory basal nucleus: *d =* 0.97, *p* = 0.04; Figure 4). Most other structures demonstrated atrophy from Braak stage III‐IV or from Braak stage V‐VI (DG and the anterior amygdaloid area) onward. We did not observe significant atrophy in parasubiculum, fimbria, and hippocampal fissure at Braak stage V‐VI.

**FIGURE 5:**
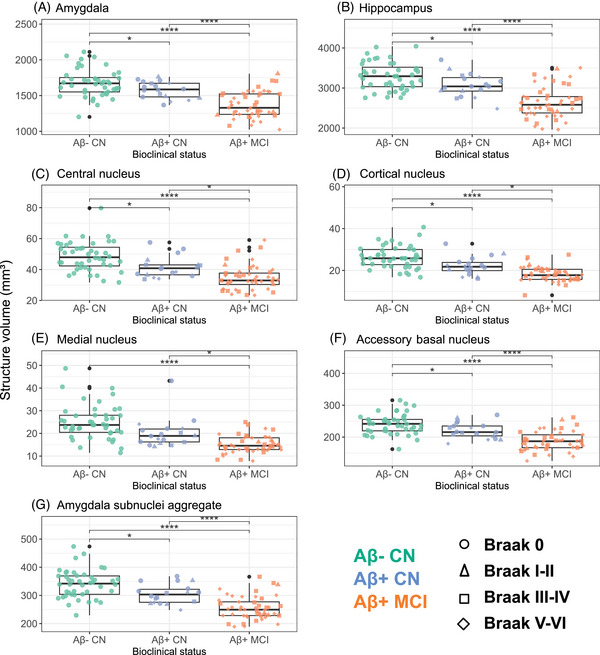
Volume difference according to amyloid and clinical classification (global structures and amygdala subnuclei). Boxplots illustrate volume differences according to bioclinical status (including Aβ and neuropsychological status) in subjects from UCLouvain cohort. The structures examined include the amygdala, the hippocampus, four amygdala subnuclei (cortical, central, medial, and accessory‐basal nuclei), and the previously defined AA. Individual data points are differentiated according to bioclinical status (color) and Braak visual stage (shape). Reported *p* values are *p* values adjusted for age, sex, intracranial volume, and years of education: ^*^ *p* < 0.05; ^**^ *p* < 0.01; ^***^ *p* < 0.001; ^****^ *p* < 0.0001. AA, amygdala aggregate.

**FIGURE 6:**
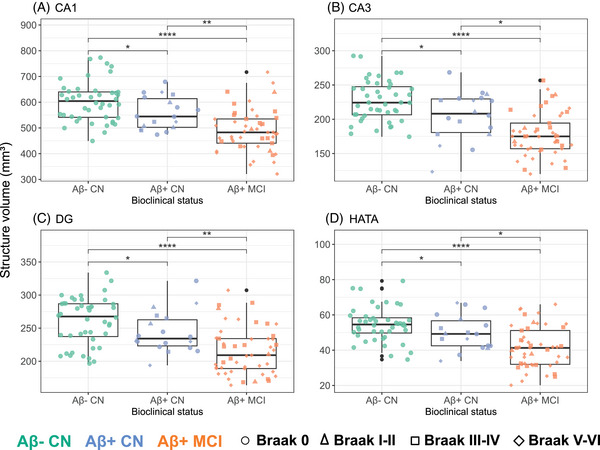
Volume difference according to amyloid and clinical classification (hippocampal subfields). Boxplots illustrate volume differences according to bioclinical status (including Aβ and neuropsychological status) in subjects from UCLouvain cohort. The structures examined include hippocampal subfields as CA1, CA3, the DG, and the HATA. Individual data points are differentiated according to bioclinical status (color) and Braak visual stage (shape). Reported *p* values are *p* values adjusted for age, sex, intracranial volume, and years of education: ^*^ *p* < 0.05; ^**^ *p* < 0.01; ^***^ *p* < 0.001; ^****^ *p* < 0.0001. DG, dentate gryrus; HATA, hippocampal amygdaloid transition area.

We finally compared the volumetric data between bioclinical groups (Aβ− CN vs Aβ+ CN vs Aβ+ MCI). The volume of the AA (*d =* 0.80, *p* = 0.01), its four substructures (central nucleus: *d = *0.65, *p* = 0.05; cortical nucleus: *d = *0.76, *p* = 0.02; medial nucleus: *d = *0.61, *p* = 0.05; accessory basal nucleus: *d = *0.74, *p* = 0.02), the global hippocampus (*d =* 0.70, *p* = 0.03), and global amygdala (*d =* 0.62, *p* = 0.05; Figure 5), but also CA1 (*d =* 0.77, *p* = 0.02), CA3 (*d =* 0.74, *p* = 0.02), DG (*d =* 0.65, *p* = 0.04), and HATA (*d =* 0.63, *p* = 0.05; Figure 6) were significantly reduced in Aβ+ CN compared with Aβ− CN. All other structures showed atrophy in Aβ+ MCI only, except the parasubiculum, the fimbria, and the hippocampal fissure, which demonstrated no atrophy.

## 4 DISCUSSION

In this study, we aimed to evaluate the association between volumetric data of MTL substructures and tau‐PET signal in AD, with a focus on the preclinical stage. Our primary goal was to identify substructures whose volume could indicate incipient tauopathy. Using the ADNI‐3 cohort, we identified four amygdala subnuclei (central, cortical, medial, and accessory basal) whose decreased volume was associated with temporal tau in Aβ+ CN. These volumes were better explained by tauopathy than the global amygdala volume was. Using our local data, we then observed that the volume of these subnuclei distinguished individuals with Braak stages I‐II from 0, as well as Aβ+ from Aβ− CN individuals. Altogether, our results suggest that the atrophy of the central, cortical, medial, and accessory basal nuclei of the amygdala are good candidates as early biomarkers of preclinical AD and highlights the relevance of studying the amygdala in AD.

Autopsy studies have shown that the amygdala is early affected by tau pathology. In their seminal paper, Braak and Braak observed that the central and then the cortical nuclei of the amygdala display “isolated neurofibrillary tangles (NFTs),” starting at Braak stage II.^11^, ^12^ At later AD stages, additional subnuclei are affected, including the accessory basal nucleus, which shows the highest NFT burden.^44^, ^45^, ^46^ Besides tau pathology, neuronal loss (ie, atrophy) is preferentially observed in these three subnuclei compared with other amygdala nuclei.^44^, ^45^, ^46^ The level of tau tangles in the medial nucleus is debated in *post mortem* studies, with some observing early sparing by tau^11^, ^12^, ^45^, ^46^ and others suggesting that it is equally affected.^47^ However, the medial nucleus is highly affected by neuritic plaques containing both Aβ and tau.^46^, ^47^ In addition, *post mortem* MRI studies observed a high density of NFTs in the basal region of the amygdala (corresponding to both the accessory basal and the basal nuclei when using FreeSurfer ROIs) in both early^48^ and advanced^49^ AD. Taken together, neuropathology and *post mortem* MRI data indicate that the amygdala is early affected in AD, particularly the cortical and basal amygdala, corresponding well to the subnuclei we identified as associated with tau in preclinical AD. In contrast, the lateral and anterior amygdala seems to be more preserved.

Previous in vivo MRI studies mostly focused on hippocampal subfields, probably because the segmentation of amygdala subnuclei has proven more difficult and was only made recently available in automated segmentation software. The association between tau pathology and MTL atrophy is well documented, including in the entorhinal cortex,^50^ the CA subfields, and the subiculum within the hippocampus.^51^ However, these studies focused on the symptomatic stages of AD. In preclinical AD, a temporal tau‐PET signal is associated with longitudinal hippocampal atrophy.^7^, ^16^ Among hippocampal subfields, the volume of the presubiculum is most associated with Aβ pathology and subsequent cognitive decline.^24^ However, to the best of our knowledge, no previous study evaluated the association between tau‐PET and hippocampal subfield atrophy in preclinical AD. We observed no significant associations between hippocampus volume or its subfields and temporal tau. However, we observed hippocampal atrophy, specifically in CA1, CA3, DG, and HATA subfields in Aβ+ CN individuals, but not in individuals with early tau pathology (Braak I‐II). This might be because tau pathology is not the only driver of hippocampal atrophy in preclinical AD as hippocampal atrophy is also observed with vascular pathology^52^ and LATE,^15^ which are both associated with Aβ independently of tau pathology.

A limited number of MRI studies investigated amygdala volume and observed atrophy from the MCI stage,^25^, ^26^, ^28^ specifically in the accessory basal nuclei.^25^, ^53^ The accessory basal, the central, and the medial nuclei have also been observed to be atrophic in CN individuals progressing to MCI,^49^ which is consistent with our observation of atrophy in these subnuclei with increasing tau‐PET signal. Longitudinal MRI studies observed sequential regional atrophy within the MTL: first in the entorhinal, then in the amygdala (specifically the accessory basal nuclei), and finally in the hippocampus (specifically CA1).^54^ Altogether, previous in vivo MRI studies highlighted the atrophy of specific amygdala subnuclei before hippocampal atrophy. The present work adds that atrophy in these amygdala subnuclei is associated with temporal tau pathology.

Interestingly, the amygdala subnuclei with early atrophy have structural connections with the earliest sites of tau pathology observed in the brain. The central nuclei is connected to the locus coeruleus,^11^ whereas the cortical and medial nuclei are connected with the olfactory bulbs.^55^ The accessory basal nucleus and the CA1 are strongly connected to the entorhinal cortex.^56^ Observing early atrophy in these subnuclei is thus consistent with a transsynaptic propagation of pathological tau.^57^

Amygdala atrophy is also observed with LATE and vascular pathology.^15^ However, there is no consensus on the specific subnuclei affected and their sequence relative to symptom onset. We cannot exclude the possibility that these pathologies contribute to the amygdala atrophy observed in our study, but the association with tau‐PET signal highlights the contribution of tau tangles. Future work will use new biomarkers, such as TDP‐43 PET, which is currently under development,^58^ to quantify the contribution of this pathology to the atrophy of specific substructures within the MTL. In the meantime, the only way to study the TDP‐43 contribution to MTL atrophy is to compare *ante mortem* MRI scans with *post mortem* measures of TDP‐43 and tau.^59^, ^60^ The studies published so far demonstrated an impact of both tau and TDP‐43 on hippocampal atrophy, but they did not examine the amygdala.

### 4.1 Limitations

Automatic amygdala segmentation is recent and has not yet been studied extensively. The absence of anatomical landmarks makes it difficult for the human eye to correct automatic segmentation in the absence of *post mortem* validation. For comparison, automatic hippocampus segmentation has been reviewed several times, following the acquisition of new *post mortem* MRI data, and is still being discussed today.^22^ It would therefore not be surprising to see the segmentation protocol evolve in the coming years, yielding different results than those observed today. The current segmentation protocol has a numerical replication rate (indicating the consistency of volume measurements upon retesting) of 67%,^61^ which clearly leaves room for improvement. However, it appears from this study in two independent cohorts that atrophy within the amygdala is not a homogeneous process at the preclinical AD stage. The use of higher‐field MRI scanners could likely improve reliability by increasing the resolution up to ∼0.44 × 0.44 mm^2^ ^62^ and better allow the distinction of small nuclei of ∼20 mm^3^.

In addition, it should be noted that the ADNI and UCLouvain datasets vary in terms of cognitive tests, language of the tests, PET tracers, and sample sizes. In particular, the strength of the associations between temporal tau burden and brain volumes, including both global and substructure volume measures for the amygdala and hippocampus, varied between the ADNI and UCLouvain datasets. We attribute these differences to a greater proportion of MCI individuals in the UCLouvain dataset as well as a great tau pathology in all clinical stages. In addition, these studies used two different tau‐PET tracers. First‐generation tau‐PET tracers such as [^18^F]Flortaucipir used in ADNI have a strong off‐target effect on melanin that notably marks the choroid plexus,^63^ while second‐generation tracers ([^18^F]MK6240) such as is used in the UCLouvain cohort, show a reduced off‐target effect.^64^ Although the hippocampus was excluded from the temporal meta‐ROI, off‐target effects might have influenced associations between tau and volumes. Finally, although we used two independent cohorts, the UCLouvain cohort contained a small number of individuals, particularly Aβ+ CN individuals. This limits statistical power and prevents us from conducting analyses in Aβ+ CN individuals only as we did in ADNI. We cannot exclude the possibility that additional regions would have demonstrated atrophy in preclinical AD or Braak stage I‐II in a larger sample.

### 4.2 Conclusions

Our study highlights the relevance of studying the amygdala in AD. By analyzing the association between amygdala subnuclei volumes and temporal tau deposition, we identified specific nuclei—central, cortical, medial, and accessory basal—that were associated with temporal tauopathy in preclinical AD. Further studies in larger cohorts are needed to confirm these results and determine whether these nuclei could be good candidates as early biomarkers for preclinical AD. These findings are supported by both traditional neuropathological and neuroimaging studies. While our results demonstrate an association between temporal tauopathy and amygdala atrophy, they also raise questions about the potential contribution of other pathologies, such as TDP‐43 or vascular pathology. Future studies using advanced imaging techniques and biomarkers for TDP‐43 could provide further insights into the complex relationship of different pathologies in AD progression.

## CONFLICT OF INTEREST STATEMENT

The firm Lantheus Inc. supplied the [18F]MK6240 precursor for acquiring the PET images analyzed in this article. No other conflicts of interest are reported. Author disclosures are available in the supporting information.

## CONSENT STATEMENT

Informed consent was obtained from all individual participants from the UCLouvain study in accordance with the principles of the Declaration of Helsinki. Ethical approval for the study was granted by the Ethics Committee of UCLouvain (Date: May 13, 2019; Eudra‐CT number: 2018‐0034/73‐94). The ADNI study was approved by the Institutional Review Boards of all of the participating institutions. Informed written consent was obtained from all ADNI participants at each site. See www.adni‐info.org for detailed information.

## Supporting information



Supporting Information

Supporting Information

## Data Availability

Anonymized data are available on justified request to Bernard.Hanseeuw@uclouvain.be
