# Supplementary material for: Amygdala atrophies in specific subnuclei in preclinical Alzheimer's disease
Source: Alzheimers Dement. 2024 Sep 10;20(10):7205–19. doi: 10.1002/alz.14235 (PMC11485073; doi:10.1002/alz.14235)
Supplement: Supplementary file 1 — Supporting Information [file ALZ-20-7205-s002.docx]

**Amygdala atrophies in specific subnuclei in preclinical Alzheimer's disease**

Supplementary material 1 : R² and p-value for all covariates of the partial Spearman correlations in the ADNI cohort.

| **ADNI CN Aβ-** | | | | | |
| --- | --- | --- | --- | --- | --- |
|  | Tau SUVr  R² (p-value) | Sex  R² (p-value) | Age  R² (p-value) | IC volume  R² (p-value) | Education  R² (p-value) |
| Amygdala agregate | 0.022  (p =0.0794) | 0.001  (p =0.694) | 0.127  (p < 0.001) | 0.126  (p < 0.001) | 0.003  (p =0.539) |
| Whole Hippocampus | <0.001  (p =0.858) | 0.001  (p =0.673) | 0.172  (p < 0.001) | 0.197  (p < 0.001) | <0.001  (p =0.999) |
| Whole Amygdala | <0.001  (p =0.989) | 0.011  (p =0.0752) | 0.19  (p < 0.001) | 0.2  (p < 0.001) | 0.004  (p =0.293) |
| Cortical nucleus | <0.001  (p =0.887) | 0.007  (p =0.145) | 0.185  (p < 0.001) | 0.01  (p =0.0838) | <0.001  (p =0.989) |
| Central nucleus | <0.001  (p =0.706) | 0.006  (p =0.177) | 0.182  (p < 0.001) | 0.054  (p < 0.001) | <0.001  (p =0.871) |
| Medial nucleus | <0.001  (p =0.929) | 0.005  (p =0.213) | 0.148  (p < 0.001) | 0.001  (p =0.555) | 0.002  (p =0.424) |
| Accessory basal nucleus | <0.001  (p =0.779) | 0.006  (p =0.167) | 0.22  (p < 0.001) | 0.137  (p < 0.001) | <0.001  (p =0.776) |
| **ADNI CN Aβ+** | | | | | |
|  | Tau SUVr  R² (p-value) | Sex  R² (p-value) | Age  R² (p-value) | IC volume  R² (p-value) | Education  R² (p-value) |
| Amygdala agregate | 0.022  (p =0.0794) | 0.001  (p =0.694) | 0.127  (p < 0.001) | 0.126  (p < 0.001) | 0.003  (p =0.539) |
| Whole Hippocampus | <0.001  (p =0.985) | 0.002  (p =0.582) | 0.172  (p < 0.001) | 0.231  (p < 0.001) | 0.012  (p =0.194) |
| Whole Amygdala | 0.001  (p =0.73) | 0.016  (p =0.133) | 0.128  (p < 0.001) | 0.209  (p < 0.001) | 0.021  (p =0.0913) |
| Cortical nucleus | 0.024  (p =0.0677) | 0.001  (p =0.732) | 0.136  (p < 0.001) | 0.032  (p =0.0359) | 0.001  (p =0.792) |
| Central nucleus | 0.023 (p =0.0719) | 0.001  (p =0.714) | 0.095  (p < 0.001) | 0.092  (p < 0.001) | <0.001  (p =0.972) |
| Medial nucleus | 0.02  (p =0.0974) | 0.001  (p =0.652) | 0.075  (p =0.00104) | 0.017  (p =0.125) | <0.001  (p =0.932) |
| Accessory basal nucleus | 0.014  (p =0.159) | 0.009  (p =0.276) | 0.131  (p < 0.001) | 0.159  (p < 0.001) | 0.004  (p =0.442) |
| **ADNI MCI Aβ+** | | | | | |
|  | Tau SUVr  R² (p-value) | Sex  R² (p-value) | Age  R² (p-value) | IC volume  R² (p-value) | Education  R² (p-value) |
| Amygdala agregate | 0.089  (p < 0.001) | 0.001  (p =0.483) | 0.183  (p < 0.001) | 0.076  (p < 0.001) | 0.003  (p =0.187) |
| Whole Hippocampus | 0.145  (p < 0.001) | < 0.001  (p =0.987) | 0.139  (p < 0.001) | 0.163  (p < 0.001) | 0.006  (p =0.38) |
| Whole Amygdala | 0.137  (p < 0.001) | < 0.001  (p =0.852) | 0.135  (p < 0.001) | 0.162  (p < 0.001) | 0.008  (p =0.324) |
| Cortical nucleus | 0.086  (p < 0.001) | 0.002  (p =0.646) | 0.2  (p < 0.001) | 0.028  (p =0.061) | 0.001  (p =0.664) |
| Central nucleus | 0.08  (p =0.00123) | < 0.001  (p =0.905) | 0.235  (p < 0.001) | 0.041  (p =0.0225) | 0.002  (p =0.606) |
| Medial nucleus | 0.021  (p =0.106) | 0.005  (p =0.444) | 0.212  (p < 0.001) | 0.017  (p =0.141) | 0.001  (p =0.757) |
| Accessory basal nucleus | 0.151  (p < 0.001) | < 0.001  (p =0.878) | 0.182  (p < 0.001) | 0.114  (p < 0.001) | 0.005  (p =0.416) |
| **ADNI All** | | | | | |
|  | Tau SUVr  R² (p-value) | Sex  R² (p-value) | Age  R² (p-value) | IC volume  R² (p-value) | Education  R² (p-value) |
| Amygdala agregate | 0.089  (p < 0.001) | 0.001  (p =0.483) | 0.183  (p < 0.001) | 0.076  (p < 0.001) | 0.003  (p =0.187) |
| Whole Hippocampus | 0.072  (p < 0.001) | < 0.001  (p =0.784) | 0.147  (p < 0.001) | 0.157  (p < 0.001) | 0.003  (p =0.189) |
| Whole Amygdala | 0.076  (p < 0.001) | 0.004  (p =0.14) | 0.146  (p < 0.001) | 0.157  (p < 0.001) | < 0.001  (p =0.634) |
| Cortical nucleus | 0.064  (p < 0.001) | 0.002  (p =0.349) | 0.159  (p < 0.001) | 0.011  (p =0.0122) | 0.003  (p =0.158) |
| Central nucleus | 0.07  (p < 0.001) | < 0.001  (p =0.845) | 0.159  (p < 0.001) | 0.043  (p < 0.001) | 0.004  (p =0.13) |
| Medial nucleus | 0.042  (p < 0.001) | 0.001  (p =0.579) | 0.134  (p < 0.001) | 0.004  (p =0.138) | < 0.001  (p =0.694) |
| Accessory basal nucleus | 0.088  (p < 0.001) | 0.002  (p =0.334) | 0.165  (p < 0.001) | 0.107  (p < 0.001) | 0.003  (p =0.174) |

To study the association between tau burden in the temporal lobe and the volumes of different (sub)structures, we performed Spearman correlations adjusted for age, sex, intracranial volume, and years of education. We report here all the Spearman coefficients between volumes and covariates, as well as the associated p-values, in the different groups of the ADNI cohort.

Supplementary material 2 : R² and p-value for all covariates of the partial Spearman correlations in the UCLouvain cohort.

| **UCLouvain CN Aβ-** | | | | | |
| --- | --- | --- | --- | --- | --- |
|  | Tau SUVr  R² (p-value) | Sex  R² (p-value) | Age  R² (p-value) | IC volume  R² (p-value) | Education  R² (p-value) |
| Amygdala agregate | 0.193  (p =0.152) | 0.329  (p =0.0512) | 0.324  (p =0.0533) | 0.005  (p =0.833) | 0.055  (p =0.463) |
| Whole Hippocampus | 0.043  (p =0.515) | 0.004  (p =0.848) | 0.165  (p =0.19) | 0.003  (p =0.868) | 0.065  (p =0.422) |
| Whole Amygdala | 0.002  (p =0.893) | 0.043  (p =0.519) | 0.235  (p =0.111) | 0.004  (p =0.852) | 0.12  (p =0.27) |
| Cortical nucleus | 0.148  (p =0.217) | 0.126  (p =0.258) | 0.033  (p =0.574) | 0.172  (p =0.181) | 0.001  (p =0.914) |
| Central nucleus | 0.036  (p =0.552) | 0.068  (p =0.413) | 0.095  (p =0.329) | 0.001  (p =0.925) | 0.008  (p =0.776) |
| Medial nucleus | 0.208  (p =0.136) | 0.244  (p =0.103) | 0.002  (p =0.894) | 0.15  (p =0.213) | 0.316  (p =0.0572) |
| Accessory basal nucleus | 0.081  (p =0.369) | 0.187  (p =0.161) | 0.434  (p =0.0199) | <0.001  (p =0.964) | 0.285  (p =0.0741) |
| **UCLouvain CN Aβ+** | | | | | |
|  | Tau SUVr  R² (p-value) | Sex  R² (p-value) | Age  R² (p-value) | IC volume  R² (p-value) | Education  R² (p-value) |
| Amygdala agregate | 0.283  (p =0.092) | 0.493  (p =0.016) | 0.41  (p =0.034) | 0.045  (p =0.533) | 0.076  (p =0.411) |
| Whole Hippocampus | 0.057  (p =0.480) | 0.027  (p =0.627) | 0.182  (p =0.19) | 0.028  (p =0.621) | 0.071  (p =0.429) |
| Whole Amygdala | < 0.001  (p =0.972) | 0.099  (p =0.345) | 0.261  (p =0.11) | 0.009  (p =0.785) | 0.132  (p =0.272) |
| Cortical nucleus | 0.267  (p =0.103) | 0.368  (p =0.048) | 0.069  (p =0.43) | 0.016  (p =0.712) | 0.001  (p =0.922) |
| Central nucleus | 0.04  (p =0.556) | 0.074  (p =0.418) | 0.098  (p =0.35) | 0.005  (p =0.837) | 0.009  (p =0.785) |
| Medial nucleus | 0.341  (p =0.059) | 0.483  (p =0.018) | 0.009  (p =0.78) | 0.008  (p =0.788) | 0.413  (p =0.033) |
| Accessory basal nucleus | 0.122  (p =0.292) | 0.309  (p =0.076) | 0.491  (p =0.016) | 0.051  (p =0.503) | 0.327  (p =0.0662) |
| **UCLouvain MCI Aβ+** | | | | | |
|  | Tau SUVr  R² (p-value) | Sex  R² (p-value) | Age  R² (p-value) | IC volume  R² (p-value) | Education  R² (p-value) |
| Amygdala agregate | 0.03  (p =0.333) | 0.002  (p =0.809) | 0.03  (p =0.332) | 0.316  (p < 0.001) | 0.022  (p =0.408) |
| Whole Hippocampus | 0.065  (p =0.153) | 0.008  (p =0.628) | 0.114  (p =0.0544) | 0.285  (p =0.00138) | 0.014  (p =0.513) |
| Whole Amygdala | 0.013  (p =0.527) | 0.001  (p =0.873) | 0.053  (p =0.198) | 0.3  (p < 0.001) | 0.022  (p =0.408) |
| Cortical nucleus | 0.063  (p =0.16) | <0.001  (p =0.922) | 0.093  (p =0.0842) | 0.176  (p =0.015) | 0.04  (p =0.262) |
| Central nucleus | 0.025  (p =0.38) | 0.001  (p =0.854) | 0.022  (p =0.415) | 0.285  (p =0.00138) | 0.009  (p =0.59) |
| Medial nucleus | 0.034  (p =0.302) | <0.001  (p =0.925) | 0.088  (p =0.0934) | 0.055  (p =0.188) | 0.002  (p =0.8) |
| Accessory basal nucleus | 0.016  (p =0.488) | <0.001  (p =0.907) | 0.042  (p =0.255) | 0.314  (p < 0.001) | 0.037 (  p =0.287) |
| **UCLouvain All** | | | | | |
|  | Tau SUVr  R² (p-value) | Sex  R² (p-value) | Age  R² (p-value) | IC volume  R² (p-value) | Education  R² (p-value) |
| Amygdala agregate | 0.393  (p < 0.001) | < 0.001  (p =0.866) | 0.129  (p < 0.001) | 0.138  (p < 0.001) | 0.011  (p =0.307) |
| Whole Hippocampus | 0.373  (p < 0.001) | 0.001  (p =0.814) | 0.103  (p =0.00148) | 0.1  (p =0.00181) | 0.002  (p =0.653) |
| Whole Amygdala | 0.352  (p < 0.001) | < 0.001  (p =0.857) | 0.09  (p =0.00306) | 0.188  (p < 0.001) | 0.01  (p =0.343) |
| Cortical nucleus | 0.285  (p < 0.001) | < 0.001  (p =0.978) | 0.15  (p < 0.001) | 0.064  (p =0.0136) | 0.006  (p =0.463) |
| Central nucleus | 0.264  (p < 0.001) | 0.001  (p =0.806) | 0.101  (p =0.0017) | 0.096  (p =0.00227) | 0.017  (p =0.211) |
| Medial nucleus | 0.248  (p < 0.001) | 0.004  (p =0.553) | 0.096  (p =0.0023) | 0.006  (p =0.467) | 0.007  (p =0.406) |
| Accessory basal nucleus | 0.403  (p < 0.001) | < 0.001  (p =0.91) | 0.118  (p < 0.001) | 0.164  (p < 0.001) | 0.005  (p =0.485) |

To study the association between tau burden in the temporal lobe and the volumes of different (sub)structures, we performed Spearman correlations adjusted for age, sex, intracranial volume, and years of education. We report here all the Spearman coefficients between volumes and covariates, as well as the associated p-values, in the different groups of the UCLouvain cohort.
